# Lead isotopes and heavy minerals analyzed as tools to understand the distribution of lead and other potentially toxic elements in soils contaminated by Cu smelting (Legnica, Poland)

**DOI:** 10.1007/s11356-016-7655-4

**Published:** 2016-09-21

**Authors:** Rafał Tyszka, Anna Pietranik, Jakub Kierczak, Vojtěch Ettler, Martin Mihaljevič, Agnieszka Medyńska-Juraszek

**Affiliations:** 1 Department of Soil Sciences and Environmental Protection, Wrocław University of Environmental and Life Sciences, CK Norwida 25/27, 50-375 Wrocław, Poland; 2 Institute of Geological Sciences, University of Wrocław, Cybulskiego 30, 50-205 Wrocław, Poland; 3 Institute of Geochemistry, Mineralogy and Mineral Resources, Faculty of Science, Charles University in Prague, Albertov 6, 128 43 Prague 2, Czech Republic

**Keywords:** Anthropogenic Pb, Metal(oid) mobility, Legnica Cu smelter, Slags

## Abstract

Surroundings of the Legnica Cu smelter (Poland) offer insight into the behavior of Pb and other metal(oid)s in heavily contaminated soils in a relatively simple site, where lithogenic and anthropogenic Pb contributions have uniform Pb isotope composition over the time of smelter activity. Distribution of metal(oid)s decreases asymptotically with depth and below 30 cm reaches concentrations typical or lower than those of upper continental crust. Usually, such distribution is interpreted as the decrease in anthropogenic Pb contribution with depth. However, calculations based on Pb isotopes indicate that anthropogenic Pb is probably distributed both as Pb-rich particles of slags and fly ashes and Pb-poor soil solutions. Generally, anthropogenic Pb constitutes up to 100 % of Pb in the uppermost 10 cm of the soils and comes often from mechanical mixing with slag and fly ash particles as well as their weathering products. On the other hand, lower soil horizon contains anthropogenic Pb in various forms, and at depths below 30 cm, most of anthropogenic Pb comes from soil solutions and can constitute from 1 to 65 % of the Pb budget. This is consistent with secondary electron microscope (SEM) analyses of heavy mineral particles showing that, in upper horizons, Pb, Cu, and Zn are contained in various particles emitted from the smelter, which show different stages of weathering. Currently, large portion of these metals may reside in the secondary Fe-hydro-oxides. On the other hand, in deeper soil horizons, anthropogenic Pb is probably dominated by Pb coming from leaching of slag or fly ash particles. Overall, metal(oid) mobility is a dynamic process and is controlled by the soil type (cultivated versus forest) and the composition and the structure of the metal-rich particles emitted from the smelter. High proportions of anthropogenic Pb at depths below 30 cm in some soil profiles indicate that Pb (and probably other metal(oid)s) can be transported down the soil profile and the present concentration of anthropogenic Pb depends on the availability of binding sites.

**Electronic supplementary material:**

The online version of this article (doi:10.1007/s11356-016-7655-4) contains supplementary material, which is available to authorized users.

## Introduction

Base metal mining and smelting emit inorganic solids with elevated concentrations of potentially toxic elements (PTE), which are often deposited close to pollution centers (Chopin and Alloway 2007; Csavina et al. 2012). The phase and chemical composition of this fallout is often complex and, in addition to soil parameters, may control mobility of PTE within the soil (Ettler 2015 and references within). Often, the soil parameters are not correlated with metal mobility, because the metals are bonded in different phases and the phases could be decomposed under different conditions (Chrastný et al. 2012). For example, Pb emitted from some smelters is pure metallic Pb (e.g., Kierczak et al. 2013), which should undergo weathering within a few to several dozens years, similar to that observed in the soils within shooting range contaminated by Pb bullets (Cao et al. 2003; Chrastný et al. 2010). However, Pb metallic phases emitted from the smelter may occur as inclusions in other phases and, in such the case, the mobility of Pb is controlled by weathering of these phases and not the Pb phase itself. Also, physicochemical parameters of soils may vary depending on the use of soil amendments for stabilizing contaminants in soils. For example, for the Legnica smelter, pH of the soils was acidic for the first 20 years of smelter activity and, after that, it was increased by liming to 6.0–7.0 (Medyńska and Kabała 2010). This shows that each contaminated site surrounding the base metal smelter is specific and may offer different insights in the Pb mobility.

Understanding Pb mobility at such sites is important to predict behavior of Pb and other PTE and/or to plan appropriate remediation techniques. Approaches to studying such complex sites include combined analytical techniques, e.g., Pb isotopes and chemical (sequential and/or selective) extractions (e.g., Ettler et al. 2004; Fernandez et al. 2008). However, the large variety in Pb isotope composition of smelting products may cause Pb isotope variations in soils that are difficult to interpret (Fernandez et al. 2008). The aim of this study is to understand mobility of PTE around the Legnica smelter using chemical, isotope, and mineralogical analyses. The site is unique because the Pb isotope compositions of ore processed in the Legnica smelter and the produced slags seem to be similar over the time span of the smelter activity (see below, Tyszka et al. 2012). Therefore, studying the heavily contaminated soils surrounding the Legnica smelter should provide information on the mobility of Pb and other PTEs in a relatively simple site dominated by only two sources of Pb, smelter derived and natural. In such a context, we use chemical and isotope analyses of soils and EDTA extracts to understand Pb distribution in the studied soils (forest and cultivated), but we show that the full picture of Pb contamination is only obtained, when Pb-bearing phases are identified by in situ methods (e.g., SEM observations coupled with EDS and electron microprobe analyses). This approach is novel in that we show the ongoing mineral transformation and correlate them with chemical and isotope composition of soils. We are also able to identify mineral transformations, which are delayed because less reactive mineral surrounds the more reactive one. These analyses provide additional information on general processes occurring in heavily contaminated sites.

## Materials and methods

### Site description and historical background

Copper ores mined in Poland are classified as the stratabound type and occur in two major areas: the Fore-Sudetic Monocline and the North Sudetic Basin in the Lower Silesia. Historical Cu mining exploited also smaller occurrences of Cu ores, e.g., porphyry deposits associated with the emplacement of the Karkonosze Granite (e.g., Miedzianka site; Mochnacka et al. 2015). This Cu mining and smelting in SW Poland has already been active in the fourteenth century (Dziekoński 1972). In the vicinity of Miedzianka, Cu smelting in nineteenth and early twentieth centuries produced large amounts of smelting wastes, mainly pyrometallurgical slags, currently distributed within soils and sediments (Kierczak et al. 2013). The copper mining in the Fore-Sudetic Block started in 1938 and the Legnica smelter, the subject of this study, was opened in 1953. The smelter emitted fly ash materials with high metal contents, but the metal concentrations were vastly reduced in the 1980s and 1990s (Monograph of KGHM Polish Copper Company 2007).

### Sampling sites

**Fig. 1:**
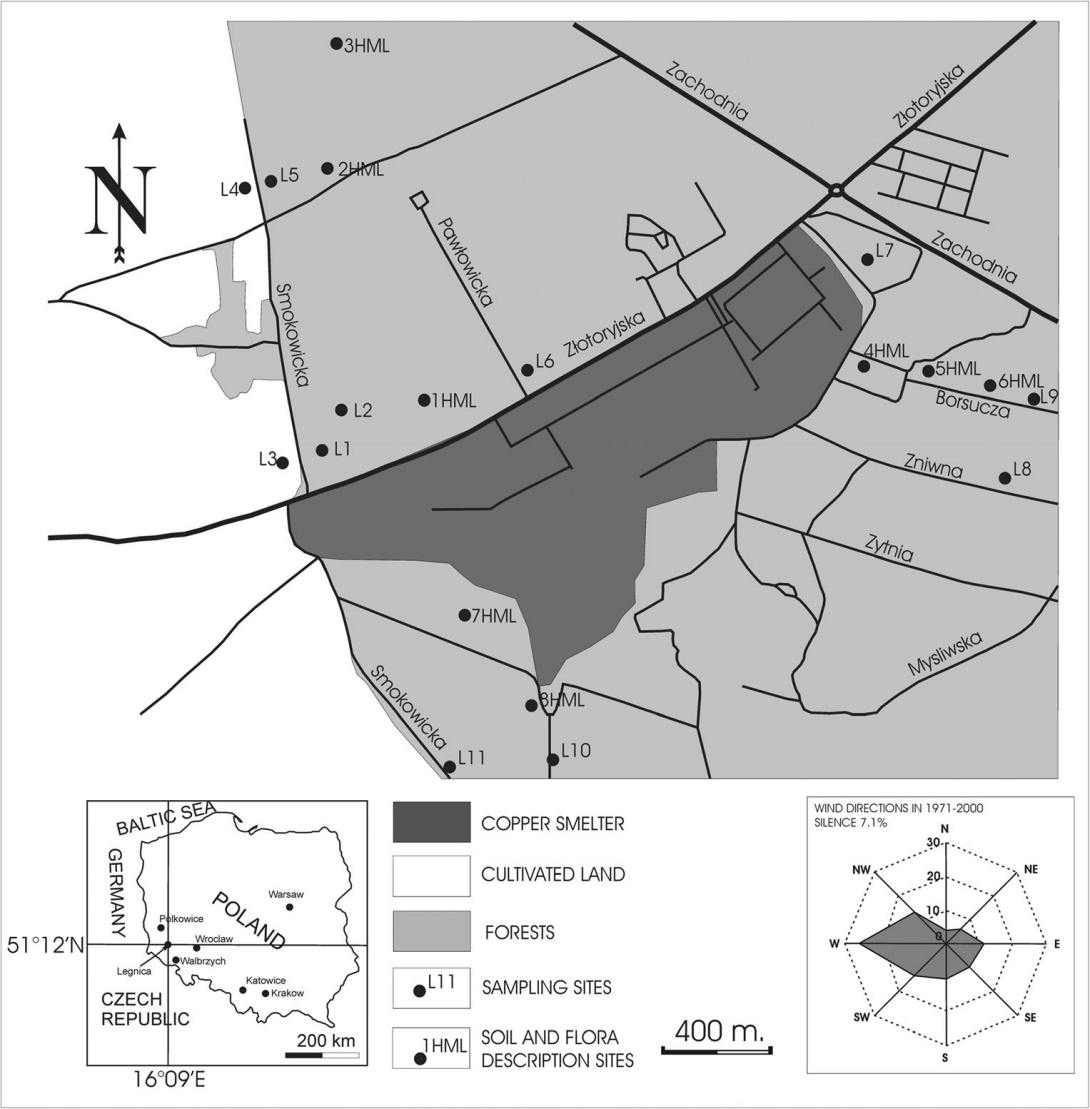
Legnica Cu smelter and its immediate surroundings with soil sampling (*L1–11*) and flora description sites (*HML1–8*) indicated. The HML sites are characterized in Supplementary material Table S1

Soils were collected from 11 profiles located around the Cu smelter in Legnica (Fig. 1). Some data on chemical and Pb isotope composition of soils from two of the profiles (L5, L7) were already presented in Tyszka et al. (2012) but interpreted only in regional and not the site-specific scale. Also, additional original data, especially on L7 profile are presented in this study. One forest soil profile (L7) and one cultivated soil profile (L4) were chosen for a detailed study.

All profiles were made in the Autumn 2009 at a distance from 100 m to 2 km from the smelter. Two pairs of the profiles were located close to each other (Fig. 1), but one from the pair was in the forest (L1 and L5) and the second on the cultivated land (L3—beetroot and L4—corn, Fig. 1; Table S1). The forested area is dominated by poplar trees, the litter is classified as mull or moder, and soils were classified as Cutanic Luvisols according to World Reference Base for Soil Resources (IUSS Working Group WRB 2014). From three to five samples were collected from each profile, and they represented (1) organic horizons, which were sampled in all the profiles in which they were developed (samples O), (2) one or two samples were taken from the horizons below the O horizons referred to as A horizons, A1 samples were always taken at the depth immediately below the O horizons (approximately 0–10 cm) and A samples were collected in the middle of each horizon (approximately 10–20 cm), (3) one or two samples were taken in the horizons underlying the A horizons (referred to as B and C horizons, approximately 30–60 cm depending on the profile). The A–C horizons were defined by the change in the color. Additionally to soil samples, four slag samples were analyzed (one collected from the L11 soil profile and three representing waste by-products from the Cu smelter). Chemical and Pb isotope data on the slag sample taken from the soil profile were partly presented in Tyszka et al. (2012).

### Samples preparation

The soil samples (approximately 3 kg) were air dried, homogenized, and sieved to <2 mm. Approximately 100 g of each sample was ground in the mortar. Fractions of 0.1 g of the ground material were dissolved in HNO_3_ and HF acids using the dissolution procedure described in Tyszka et al. (2012) in order to analyze total concentrations of metals and As and Sb in the material as well as Pb isotope compositions.

A mass of 2 g of the ground samples underwent extraction by EDTA. Extractions were carried out in 0.05 M EDTA, at a 1:10 sample to solution ratio (Quevauviller 1998). Each sample was leached in duplicate and the error for each analysis was based on analyses of these two measurements. Pb isotopes were not measured in duplicate samples.

### Analysis

#### Chemical and isotope composition of samples and leachates

The concentrations of metals (Ag, Cd, Cu, Pb, Sn, Zn) and metalloids (As, Sb) as well as the ^206^Pb/^207^Pb and ^208^Pb/^206^Pb isotope ratios in the total digests and EDTA leachates were measured by means of quadrupole-based inductively coupled plasma mass spectrometry (ICP MS; X Series 2, Thermo Scientific, Charles University, Prague). The operating conditions are described in detail in Mihaljevič et al. (2011), and the details of the analytical session of this study are given in Tyszka et al. (2012). The three slag samples (SFS, LS, GS) were analyzed after 2012, following similar procedure, however, Ag was not analyzed in these samples.

Major elements were analyzed in the samples from the L4 and L7 profiles. The analyses were carried out by the ACME Analytical Laboratory (Vancouver, Canada) by inductively coupled plasma emission spectrometry (ICP-ES) following fusion of the samples by LiBO_2_/Li_2_B_4_O_7_.

### Heavy mineral analyses

The heavy mineral fraction was separated from the soil from four samples L4A1, L4B2, L7A1, and L7B using a Na_2_WO_4_ heavy liquid. The heavy phases were mounted in polished thin sections. The sections were analyzed by Tescan Vega LSU Scanning Electron Microscope at the Wrocław University of Technology. Back-scattered electron (BSE) images of individual phases as well as corresponding EDS spectra were collected. Additionally, several quantitative analyses of heavy mineral fraction were done by the electron microprobe (Cameca SX 100, University of Warsaw) operated at an accelerating voltage of 15 kV and a beam current of 20 nA and a counting time of 10 s for all the elements except for Pb (40 s). The following standards were used for these measurements: diopside (Mg, Si, Ca), orthoclase (Al, K), albite (Na), chalcopyrite Cu, barite (S), apatite (P), hematite (Fe), rutile (Ti), rhodonite (Mn), sphalerite (Zn), and galena (Pb). Detection limits for Pb, Zn, and Cu were approximately 0.15 wt.%.

## Results

### Soil contamination around the Legnica smelter: metal(oid) concentration and Pb isotopes

**Fig. 2:**
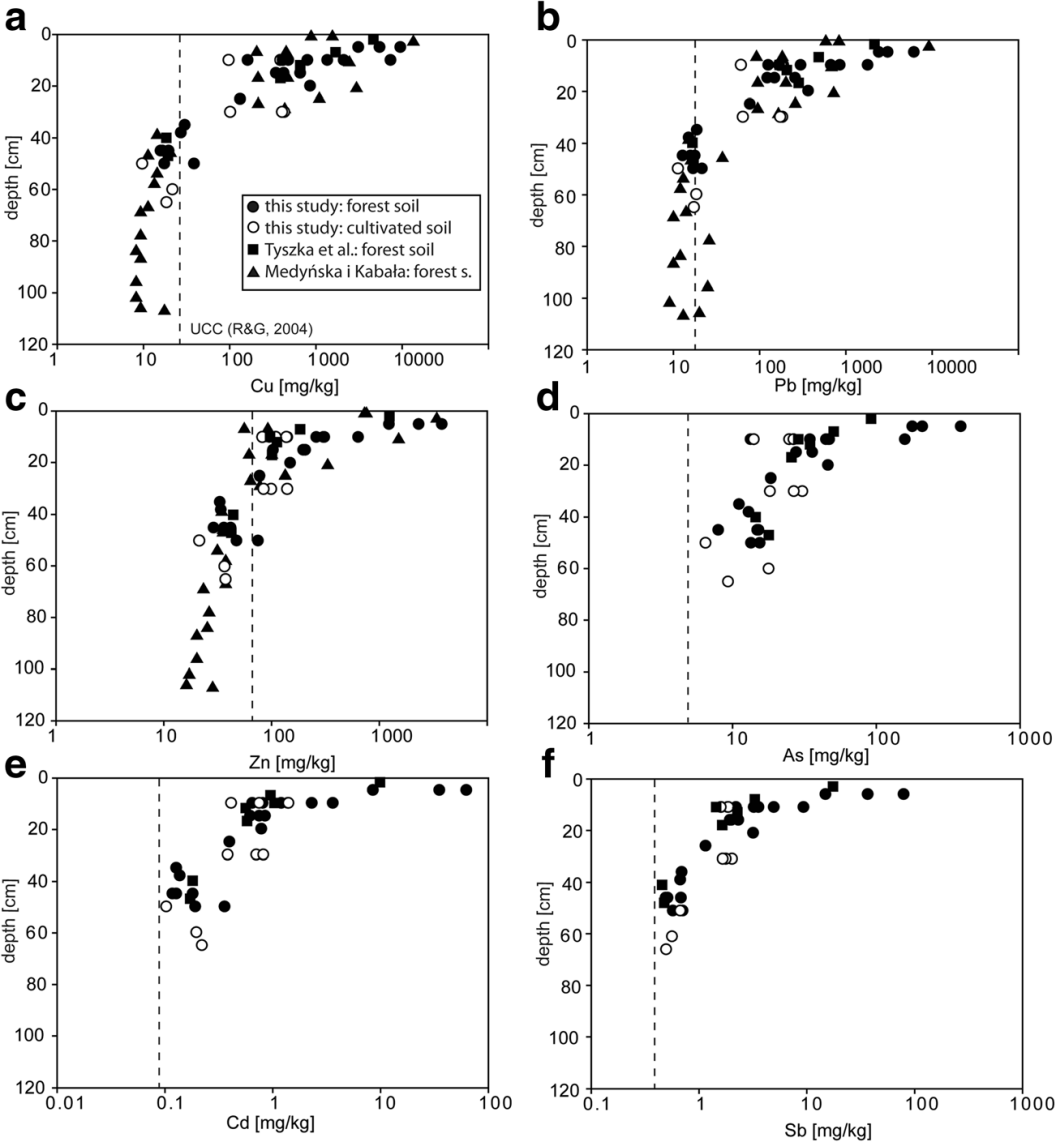
Distribution of potentially toxic elements with depth in soils located around Legnica smelter. The *dashed line* represents the average upper crust composition after Rudnick and Gao (2004)

Despite the significant decrease in pollution output from the Legnica smelter over 20 years ago, the soils around the smelter still exhibit high concentrations of metals and metalloids (Karczewska et al. 2010). It is because the mobility of metals is reduced in soils with alkaline pH, which prevail around the Legnica smelter (Karczewska et al. 2010; Medyńska and Kabała 2010). In more acidic conditions, the mobility of the elements may substantially increase (Medyńska and Kabała 2010) and up to 50 % of the metals may become mobile (Kabała and Signh 2001). Generally, previous research showed that soils in the vicinity of the Legnica smelter have concentrations of metals such as Cu, Pb, Zn, and Cd higher than the soil quality standards (according to the polish norm Regulation of the Minister of Environment 2002). Similarly high concentrations were found in soils collected in this study (Fig. 2; Table 1). Such high concentrations may affect crop productivity and require development of remediation techniques (e.g., Karczewska et al. 2008; Karczewska et al. 2009) and, therefore, it is vital to understand how the contamination is distributed within the soils.

**Table 1:** Chemical and Pb isotope data

Sample ID	Depth (cm)	Co (mg/kg)	Ni (mg/kg)	Cu (mg/kg)	Zn (mg/kg)	As (mg/kg)	Ag (mg/kg)	Cd (mg/kg)	Sb (mg/kg)	Sn (mg/kg)	Pb (mg/kg)	^206^Pb/^207^Pb
Soils												
L1O	5	101	38	3012	2281	175	11.5	35.9	36.0	44.6	3049	1.176
L1A1	10	18	16	464	138	34	2.8	0.7	2.2	6.4	188	1.180
L1A	15	28	16	334	102	27	2.2	0.6	1.9	6.1	149	1.185
L1B	35	19	14	29	33	11	0.8	0.1	0.7	1.8	19	1.198
L1C	45	39	25	16	36	8	0.7	0.1	0.5	1.7	13	1.206
L2O	5	140	51	5313	3742	380	21.3	63.9	77.5	98.9	6149	1.177
L2A1	10	24	17	772	256	44	3.1	1.2	3.2	7.4	298	1.174
L2A	15	35	14	410	202	27	2.2	0.8	2.0	3.6	122	1.185
L2B	38	19	11	26	33	13	1.1	0.1	0.7	1.7	15	1.208
L3A1	10	18	14	376	135	24	2.5	0.8	1.8	5.3	168	1.180
L3B	30	19	13	415	138	30	2.7	0.7	2.0	5.5	182	1.182
L3C	65	39	24	18	37	9	1.2	0.2	0.5	2.0	17	1.213
L4A1	10	18	16	400	107	26	2.6	1.4	1.8	4.7	176	1.181
L4B	30	16	12	393	98	26	2.7	0.8	1.7	4.5	173	1.178
L4C	60	14	16	21	36	18	1.3	0.2	0.6	2.1	18	1.216
L5A1^a^	10	21	14	*393*	*95*	*28*	2.5	*1.1*	1.4	4.2	*170*	*1.183*
L5A^a^	17	16	13	*376*	*100*	*25*	2.5	*0.6*	1.6	3.9	*284*	*1.178*
L5B^a^	47	12	22	*19*	*42*	*18*	1.2	*0.2*	0.5	2.1	*17*	*1.225*
L6O	5	58	39	9243	1209	205	28.3	8.7	14.6	27.9	2403	1.176
L6A	10	46	32	7123	626	155	23.5	3.7	9.1	23.3	1786	1.184
L6B	45	24	11	15	29	15	1.1	0.1	0.5	1.8	18	1.217
L7O^a^	2	136	69	*4507*	*1222*	*90*	18.5	*10.1*	17.1	28.8	2146	*1.179*
L7A1^a^	7	29	21	*1640*	*181*	*50*	5.9	*1.0*	3.2	10.8	484	*1.173*
L7A^a^	12	20	15	*640*	*110*	*34*	3.3	*0.6*	2.2	6.0	207	*1.181*
L7B^a^	40	19	19	*18*	*44*	*14*	1.2	*0.2*	0.4	2.0	17	*1.218*
L8O	10	27	22	1316	298	46	6.1	2.4	4.8	11.9	668	1.177
L8A	20	32	18	832	146	45	4.8	0.8	3.1	8.5	365	1.178
L8B	50	19	21	37	74	13	1.3	0.4	0.6	2.0	21	1.199
L9A1	10	25	20	2042	304	46	7.7	1.4	3.5	12.0	849	1.176
L9A	15	30	16	638	194	35	3.1	0.9	2.3	5.8	258	1.180
L9B	45	21	14	19	41	15	1.3	0.2	0.7	1.8	16	1.211
L10O	10	50	12	157	94	13	1.2	0.8	1.6	3.1	125	1.181
L10A	25	40	13	128	76	18	2.3	0.4	1.1	2.7	77	1.185
L10B	50	21	16	17	47	15	1.5	0.2	0.7	1.8	17	1.211
L11A1	10	22	16	94	81	14	1.2	0.4	1.6	2.8	61	1.177
L11B	30	32	16	98	83	18	1.1	0.4	1.6	3.4	64	1.186
L11C	50	51	7	9	21	6	0.5	0.1	0.7	1.1	11	1.185
Ores												
KGHM1^a^		72	195	*168,448*	*26*	*58*	352.0	*0.8*	1.4	3.5	*181*	*1.180*
KGHM2^a^		78	220	*100,048*	*74*	*91*	308.9	*0.9*	2.2	3.6	*7913*	*1.174*
Slags												
S115^a^		39	62	*667*	*13,299*	*1136*	20.0	*0.3*	165.7	19.2	*1642*	*1.181*
SFS		346	16	1323	2616	58	n.a.	0.8	9.1	11.0	835	1.176
LS		42	222	10,915	76,400	3569	n.a.	525.6	122.0	762.0	26,125	1.174
GS		964	357	11,425	7810	884	n.a.	4.6	39.6	22.0	21,135	1.173
Fly ashes												
HM1/4^a^		10	6	*420*	*640*	*24*	1.3	*8.2*	3.3	3.7	*490*	*1.178*
HM2/4^a^		6	5	*285*	*380*	*12*	0.9	*2.5*	1.2	0.5	*170*	*1.177*
HM3/4^a^		3	6	*120*	*360*	*3*	0.3	*2.1*	0.4	0.4	*54*	*1.178*

*n.a.* not analyzed

^a^Some element concentrations and Pb isotope ratios in the samples are reported after Tyszka et al. (2012) and presented here in italics; additional elements analyzed in the samples in this study are presented

**Fig. 3:**
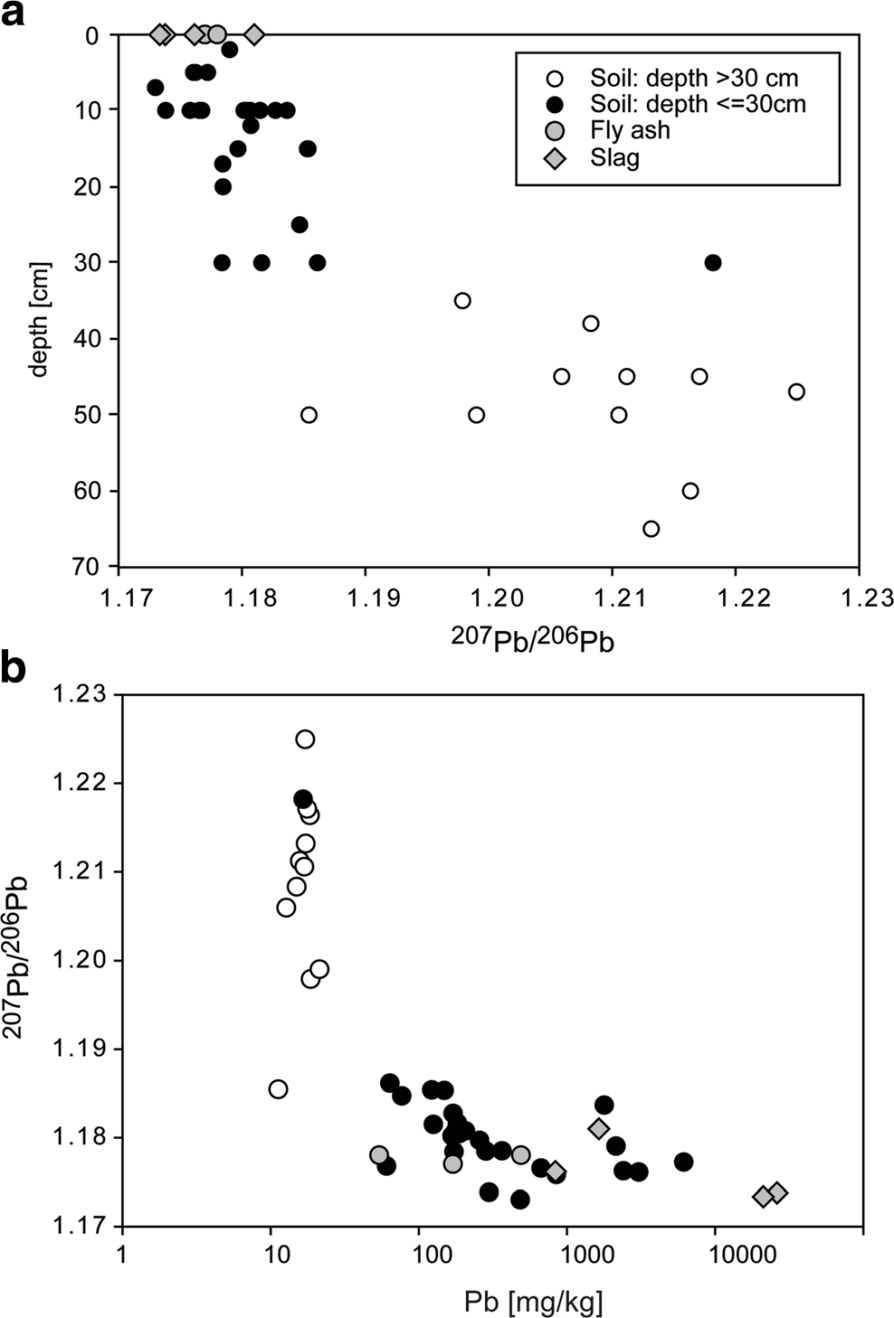
**a** Variations in Pb isotope composition with depth in soils located around Legnica Cu smelter. **b** Variations in Pb isotope composition with Pb concentration in soil samples, slags, and fly ashes

Medyńska-Juraszek and Kabała (2012) characterized metal distribution in three profiles taken in the same area as the profiles characterized in this study (Table S1). The soils in profiles have slightly acidic pH of 6.7 in the upper horizons to slightly alkaline pH of 7.7 in the lower horizons. The authors divided the soils in three horizons: horizon A, usually from 0 to 30 cm depth, horizon B (EBt, EBtg) at depths 30–80 cm, and horizon C at depths over 80 cm. Their division is similar to that presented in this study, where the change in soil properties is noticed at depths of approximately 30 cm. The comparison of the data obtained in this study (October 2009) with the soils collected in May 2009 for the study presented in Medyńska-Juraszek and Kabała (2012) show very similar distribution of Pb, Cu, and Zn (Fig. 2). Generally, metal and metalloid concentrations decrease with depth in all soils analyzed in this and Medyńska-Juraszek and Kabała’s (2012) studies (Fig. 2; Table 1). Figure 2 shows striking change in the element distribution at 30 cm, sometimes with a pronounced gap in concentrations between shallower and deeper samples (e.g., for Cu). In detail, the upper horizons (down to 30 cm) have decreasing elemental concentrations of metal and metalloids from the top to the bottom of the horizons, whereas Pb isotope composition (^206^Pb/^207^Pb ratio) is similar for all samples collected at similar depths (1.173–1.184 for depths <10 cm and 1.178–1.186 for depths between 10 and 30 cm, Fig. 3). The lower horizons (below 30 cm) have similar metal and metalloid concentrations and scattered ^206^Pb/^207^Pb value from 1.185 to 1.225 (Fig. 3). The samples at depths below 65 cm were not collected in our study, but the metal concentrations in the deeper samples collected by Medyńska-Juraszek and Kabała (2012) is similar to the samples taken at depths 30–65 cm. Only slight decrease in Pb, Cu, and Zn is observed with depth at depths 30–100 cm (Fig. 2).

### Slag composition: potential contaminant

Chemical and Pb isotope composition was analyzed in slag samples in order to compare their composition with that of soils. The assumption is that, in addition to fly ashes and ore samples, slag composition may represent typical composition of the anthropogenic material in the Legnica area. Additionally, the slag samples represent both recent (SFS, LS, GS) and past (S115) ore smelting, offering an insight into the change of isotope composition in time. Total concentrations of elements vary between the slag samples. The approximate Pb content is from 830 to 26,000 mg/kg^−1^, Zn from 2600 to 76,000 mg/kg^−1^, Cu from 670 to 11,000 mg/kg^−1^, Cd from 0.3 to 530 mg/kg^−1^, As from 60 to 3600 mg/kg^−1^, and Sb from 10 to 170 mg/kg^−1^. The highest concentrations are in the recent LS slag, only Sb is the highest in the old slag sample. On the other hand, Pb isotope composition is similar between the samples with ^206^Pb/^207^Pb ratios of 1.173–1.181, which is typical also for other smelting products (fly ashes with ^206^Pb/^207^Pb of 1.177 to 1.178) and Cu ores (1.180, Tyszka et al. 2012). These analyses support the notion that Pb isotope composition of anthropogenic material did not change during the operation time of the Legnica smelter.

### Heavy minerals: carriers of potentially toxic elements

**Fig. 4:**
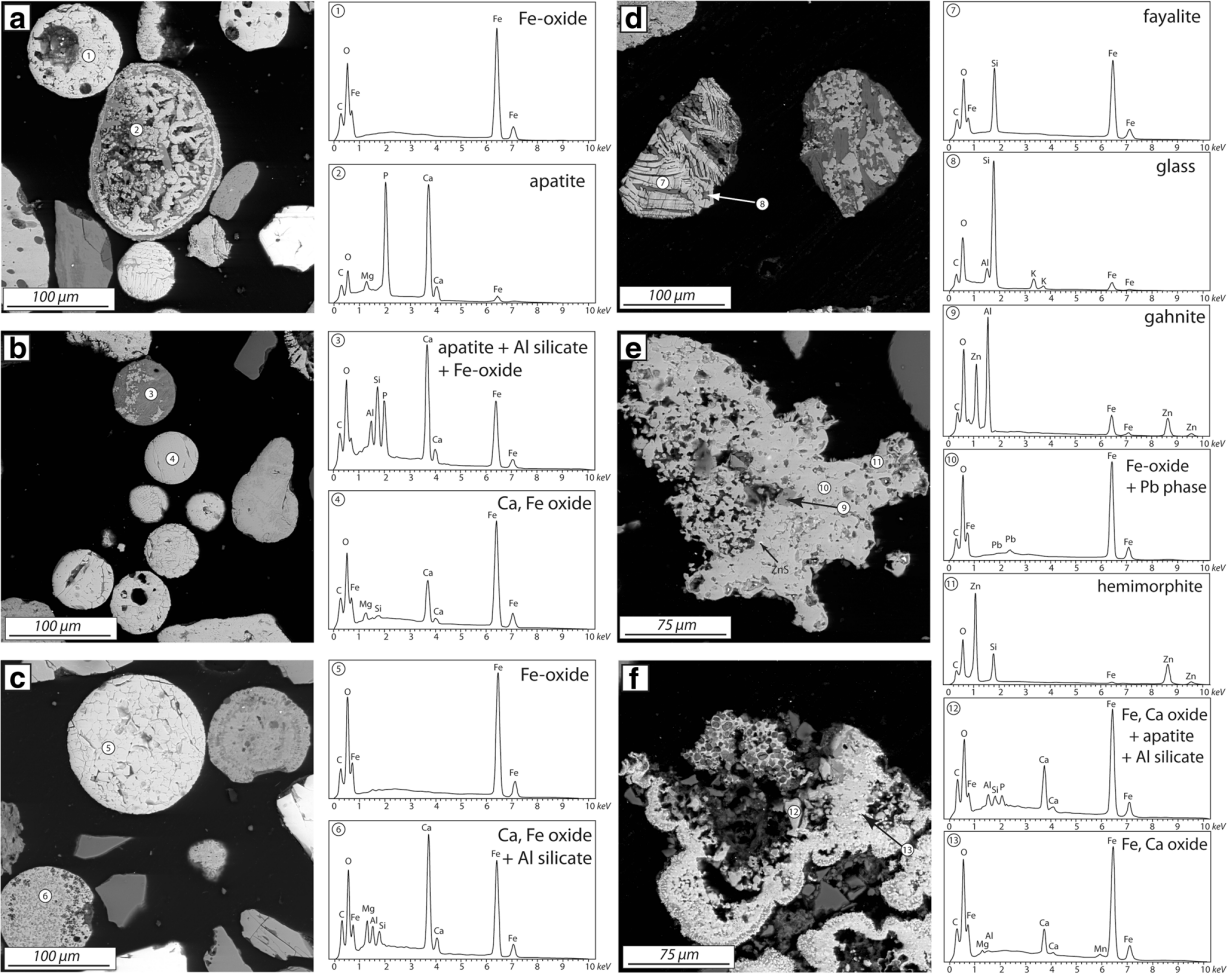
Representative heavy minerals separated from soils located around Legnica smelter: **a**, **b** cultivated soil, 10 cm depth, **c**, **e** cultivated soil, 60 cm depth, **d**, **f** forest soil, 40 cm depth

**Fig. 5:**
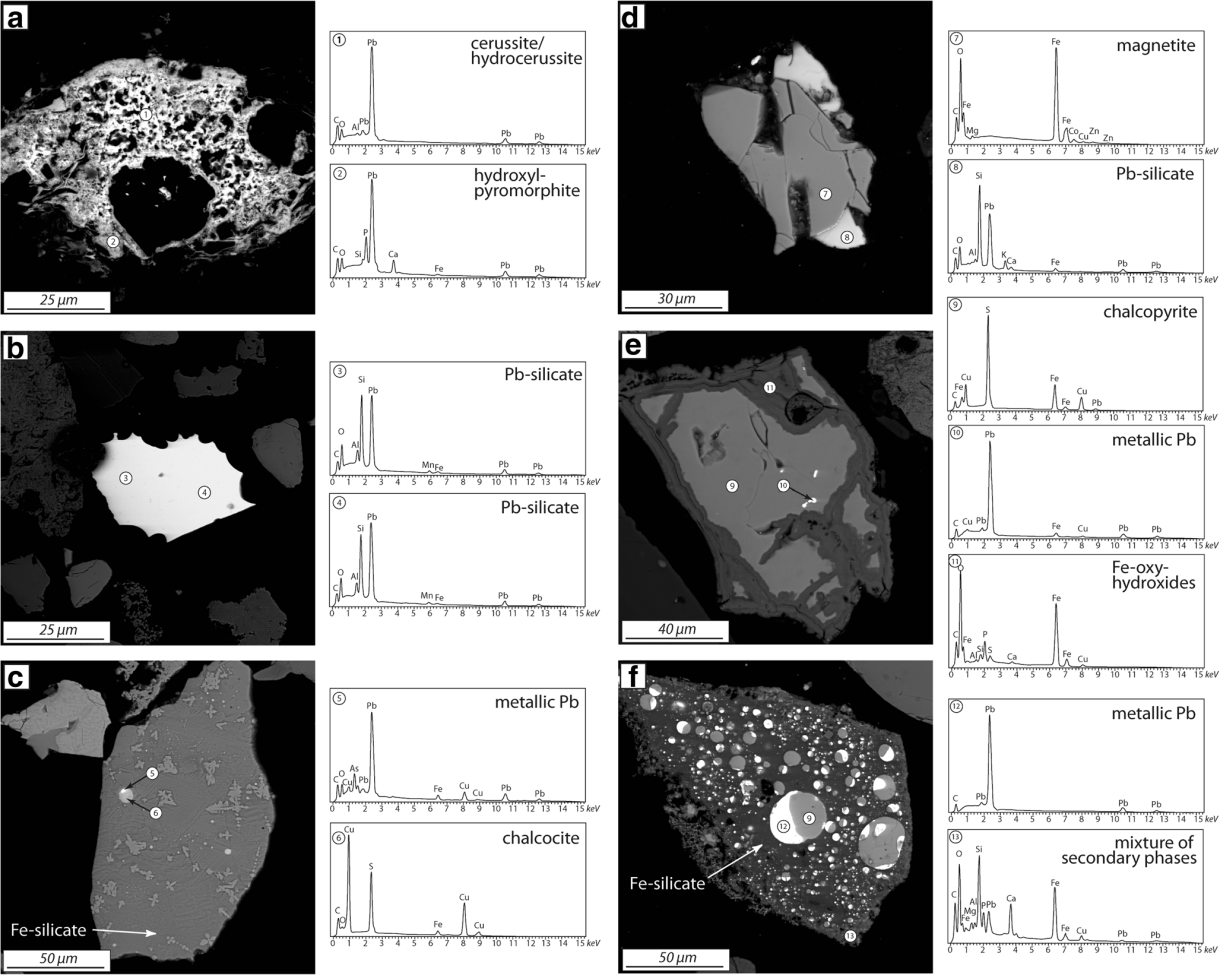
Metal-bearing heavy minerals from soils located around Legnica smelter: **a** cultivated soil, 10 cm depth, **b** cultivated soil, 60 cm depth, **c**–**f** forest soil, 12 cm depth

Observations were made also on the heavy mineral fraction separated from sites L4 (cultivated soil) and L7 (forest soil, Fig. 1). These profiles were chosen because the L7 site was the most contaminated of the forest soils and L4 represented the deepest profile from the cultivated soils. Heavy mineral analyses show that the heavy fraction is dominated by anthropogenic materials: fly ash spheres (Fig. 4a–c) and irregular slag fragments (Fig. 4d). Deeper soil horizons contain less fly ash particles (higher amounts were observed in cultivated soil than in the forest soil), but slag fragments still occur there (Fig. 4d). At lower horizons, the heavy mineral fraction of anthropogenic origin is dominated by Fe phases with irregular forms (Fig. 4 e, f), which are probably weathered remnants of ash and slag particles. Phase composition of the anthropogenic phases varies, but Fe oxides are the most common, followed by Fe–Ca oxide phase, apatite, and Fe silicate (Fig. 4). Some of the fly ash spheres are etched, usually from one side, indicating leaching of some primary phases (Fig. 4a (2), c (6)). Metals such as Cu, Zn, and Pb were detected in some heavy mineral phases in EDS spectra and also by microprobe analyses (Table 2). Copper forms mostly sulfides either chalcocite (Fig. 5c) or chalcopyrite (Fig. 5 e, f). Small amounts of Cu were also detected in Fe-oxy-hydroxides (Fig. 5e) or in the secondary minerals occurring as the rims on heavy mineral particles (Fig. 5f). Zinc occurs in irregular Fe-rich particles and forms phases such as gahnite, hemimorphite, and small inclusions of sphalerite (Fig. 4e). Pb-bearing phases comprise Pb silicate (Fig. 5 b, d) and metallic Pb (Fig. 5 c, e, f), which are probably primary phases emitted from the smelter. Metallic Pb forms small inclusions accompanied by Cu and Cu–Fe sulfides, which are often enclosed in larger silicate slag fragments (Fig. 5 c, e, f). Secondary Pb phases include cerussite (Fig. 5a (1)) and hydroxypyromorphite (Fig. 5a (2)).

**Table 2:** Microprobe analyses of Fe-rich phases from the heavy mineral fraction

	No. 1	No. 2	No. 3	No. 4	No. 5	No. 6	No. 7	No. 8	No. 9	No. 10	No. 11
SiO_2_	0.75	0.24	0.33	0.33	0.25	0.24	b.d.l.	b.d.l.	0.24	0.94	0.57
Al_2_O_3_	b.d.l.	1.51	1.66	b.d.l.	0.52	0.15	3.03	0.47	b.d.l.	b.d.l.	b.d.l.
Cr_2_O_3_	b.d.l.	b.d.l.	b.d.l.	0.54	0.27	b.d.l.	b.d.l.	b.d.l.	0.89	b.d.l.	b.d.l.
FeO	83.34	87.85	87.88	94.58	76.78	100.04	72.39	86.33	90.46	82.56	72.86
MnO	0.21	b.d.l.	b.d.l.	b.d.l.	0.45	0.43	b.d.l.	b.d.l.	0.17	b.d.l.	0.24
MgO	b.d.l.	b.d.l.	b.d.l.	b.d.l.	0.21	b.d.l.	12.27	b.d.l.	b.d.l.	b.d.l.	b.d.l.
CaO	b.d.l.	b.d.l.	0.34	b.d.l.	b.d.l.	b.d.l.	0.78	b.d.l.	b.d.l.	b.d.l.	b.d.l.
PbO	b.d.l.	b.d.l.	b.d.l.	b.d.l.	b.d.l.	b.d.l.	b.d.l.	b.d.l.	b.d.l.	0.33	0.43
CuO	0.38	0.15	0.21	b.d.l.	6.55	b.d.l.	b.d.l.	0.23	b.d.l.	0.34	1.57
ZnO	b.d.l.	b.d.l.	b.d.l.	b.d.l.	2.12	b.d.l.	b.d.l.	b.d.l.	b.d.l.	0.34	0.11
SO_3_	0.26	b.d.l.	b.d.l.	b.d.l.	b.d.l.	b.d.l.	b.d.l.	b.d.l.	b.d.l.	0.16	0.59
	84.94	89.75	90.43	95.44	87.14	100.86	88.46	87.03	91.77	84.68	76.38

Analyses 1–9 were done in primary round fly ash fragments such as those in Fig. 4a, c; analyses 10 and 11 were done in irregular Fe-rich particles interpreted as secondary in origin, such as those in Fig. 4e, f

*b.d.l.* below detection limit

Microprobe analyses show that Cu, Zn, and Pb is contained in Fe-rich spheres (such as those in Fig. 4a–d) and irregular Fe-rich phases (such as those in Fig. 4e, f; Table 2). The Zn and Cu concentration above detection limit (from 0.2 to 6.6 wt.% CuO and from 0.2 to 2.1 wt.% ZnO) were found in some Fe-rich spheres with lower totals, probably representing secondary Fe-oxy-hydroxides (Table 2). Zinc and copper occur also in irregular Fe-rich phases (0.3–1.6 wt.% CuO and 0.2–0.3 wt.% ZnO). The Pb concentration was below the EPMA detection limit in the spheres, whereas it was above the detection limit in the secondary, irregular Fe-rich particles (0.4 wt.% of PbO).

### EDTA extractions

**Fig. 6:**
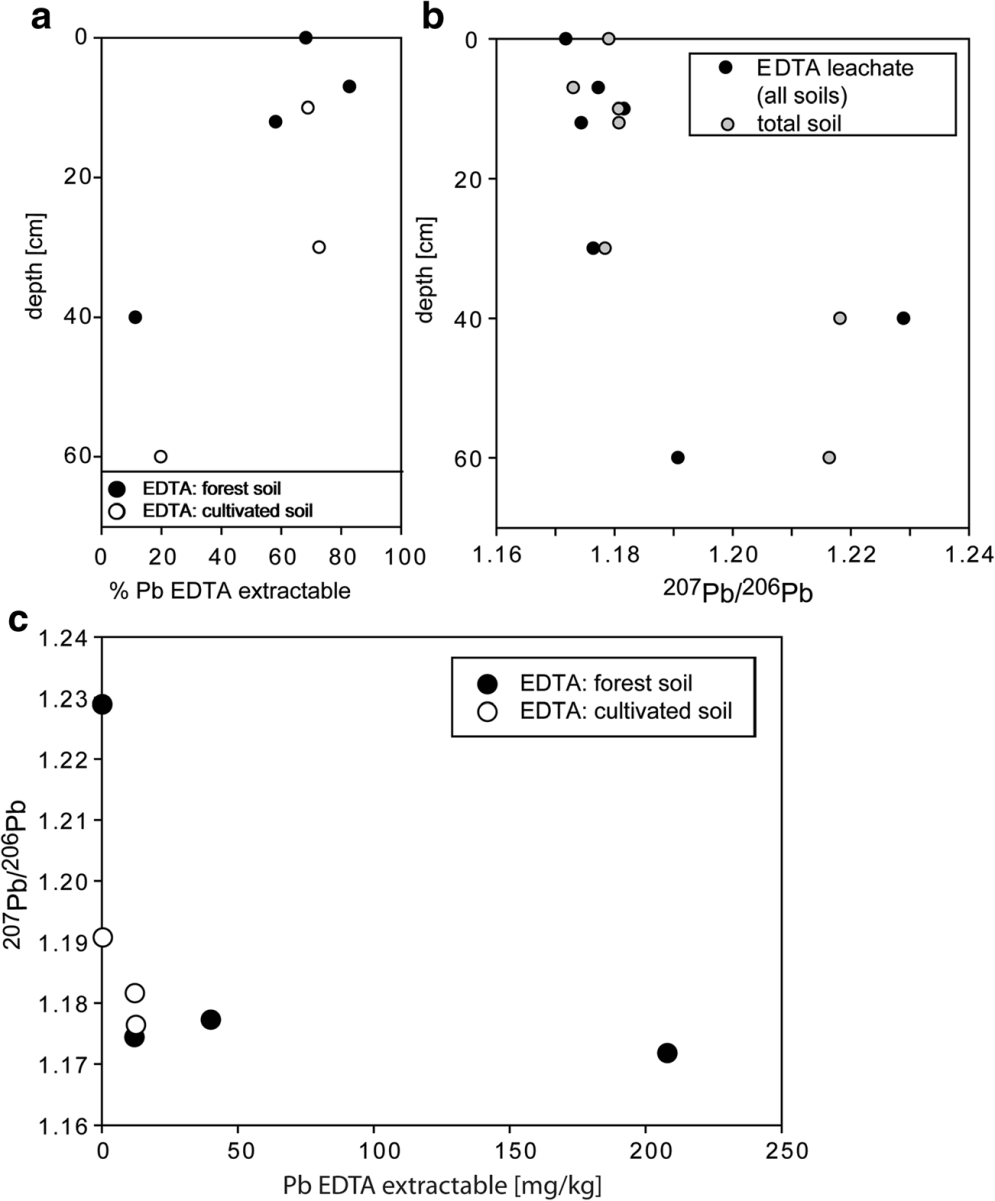
Results of EDTA leaching: **a** proportion of EDTA-extractable fraction in soil samples at different depths, **b** Pb isotope ratios in EDTA leachates and corresponding soil at different depths, **c** Pb concentration and Pb isotope ratios in EDTA leachates, comparison of forest and cultivated soils

The EDTA soil extracts were also analyzed for samples from sites L4 (cultivated soil) and L7 (forest soil, Fig. 1) for which heavy minerals were analyzed. Generally, the concentrations of EDTA extractable elements decreased from top to the bottom of the soil profiles (Table 3; Fig. 6a). The organic horizon at the site L7 showed the highest EDTA extractable concentrations for Cu, Zn, and As, but concentrations of Pb, Cd, and Sb were lower than those extracted from the A horizon (Table 3). The proportion of extractable metal(oid)s was similar between the cultivated and forest soils at 10 cm, but differed at depths 30 cm and below, with cultivated soils having higher EDTA extractable proportions than those from the forest soils (Fig. 6a). In terms of Pb isotope composition, the cultivated soil has similar Pb ratios at all soil depths, whereas the forest soil has lower ratios in upper soil horizons than in the lower horizon (Fig. 6b, c). Usually, the Pb isotope composition of the leachate was similar or slightly lower than the bulk soil (Fig. 6b).

**Table 3:** Analyses of EDTA-extractable fraction

Sample	Depth	EDTA Pb		EDTA Cu		EDTA Zn		EDTA As		EDTA Cd		EDTA Sb		EDTA ^206^Pb/^207^Pb
		mg/kg	%	mg/kg	%	mg/kg	%	mg/kg	%	mg/kg	%	mg/kg	%	
L7O	0	208 ± 3	68	393 ± 12	87	79 ± 3	64	1.6 ± 0.1	18	0.8 ± 0.06	22	0.04 ± 0.00	1.1	1.172
L7A1	7	40 ± 3	82	105 ± 13	64	4.9 ± 0.1	27	0.30 ± 0.05	6.3	0.05 ± 0.00	52	0.02 ± 0.00	6.1	1.177
L7A	12	12 ± 0	58	34 ± 1	53	2.6 ± 0.0	24	0.08 ± 0.01	2.4	0.03 ± 0.00	45	0.01 ± 0.00	5.7	1.174
L7B	40	0.2 ± 0.1	11	0.3 ± 0.0	14	0.1 ± 0.0	1.9	0.01 ± 0.00	0.7	b.d.l.	–	b.d.l.	–	1.230
L4A1	10	12 ± 1	68	25 ± 1	63	2.8 ± 0.4	26	0.3 ± 0.05	10	0.05 ± 0.01	32	0.02 ± 0.00	8.8	1.182
L4B	30	13 ± 1	72	26 ± 3	66	3 ± 0.2	31	0.3 ± 0.04	12	0.05 ± 0.00	58	0.02 ± 0.00	11	1.176
L4C	60	0.4 ± 0.02	19	0.5 ± 0.01	25	0.1 ± 0.00	2.4	b.d.l.	–	b.d.l.	–	b.d.l.	–	1.191

Each result for trace elements is the average of two analyses (2SD is shown for each measurement). Pb isotopes were measured in one leachate.

% shows proportion of leached metal(oid)s concentration to total metal(oid)s concentration

## Discussion

### Metal(oid)s and Pb isotope distribution in soils: implications on origin and mobility of contaminants

The soils located within the few-kilometer zone around the Legnica Cu smelter have typical characteristic of soils heavily polluted by atmospheric fallout. They show asymptotic decrease in Pb and other PTE concentrations characterized by rapid decrease in the upper soil horizons (first 30 cm) and approximately constant values in the lower soil horizons, close to the reference value for the upper continental crust (Fig. 2). Similar Pb distribution was observed in many contaminated soils worldwide (e.g., Watmough et al. 2004, Walraven et al. 2014) and was associated with an atmospheric contamination source, e.g., gasoline (Kuang et al. 2013; Walraven et al. 2014) or ore smelting (e.g., Chopin and Alloway 2007; Prapaipong et al. 2008). Also, the metal(oid) concentration decreases with the increasing distance from the smelter, which is typical for the contamination dominated by a single source (e.g., Roszyk and Szerszeń 1988; Prapaipong et al. 2008; Medyńska and Kabała 2010).

These elevated concentrations of Pb and other metal(oid)s in upper soil horizons compared with lower soil horizon in the soils surrounding Legnica smelter seem to indicate that the contamination is concentrated or limited to the first 30 cm of the soil. Also, the same patterns for relatively immobile Pb and mobile Zn suggest that the contamination was successfully immobilized within the upper soil horizons, probably by introducing practices aiming at preservation of neutral to slightly alkaline pH in the studied soils (Medyńska-Juraszek and Kabała 2012).

**Fig. 7:**
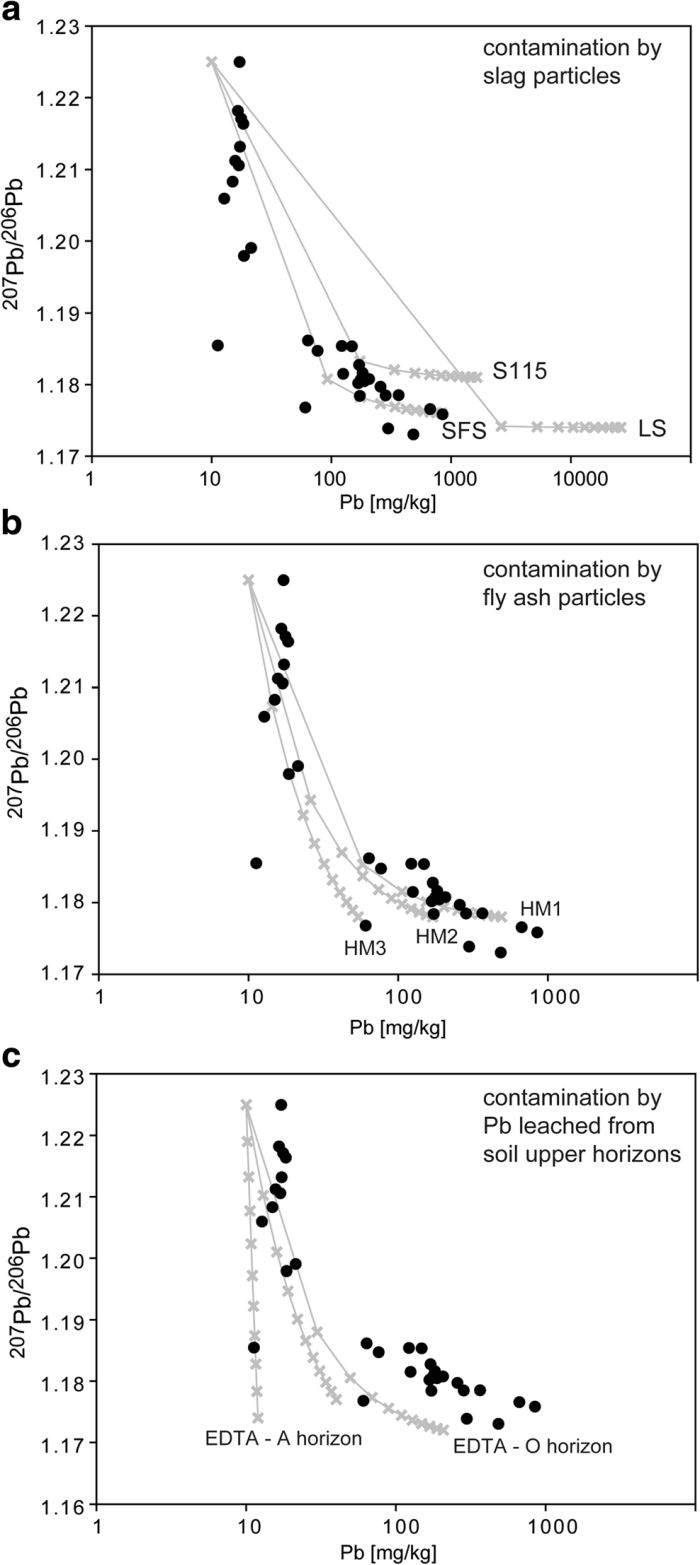
A simple two-component mixing models between sources with different Pb concentrations and Pb isotope ratios. The model assumes one lithogenic Pb source with uniform Pb isotope ratio and Pb concentration and variety of potential anthropogenic Pb sources such as **a** slags, **b** fly ashes, and **c** EDTA leachates with compositional range taken from samples analyzed in this or previous studies (Tyszka et al. 2012). Lithogenic Pb can be taken as uniform (^206^Pb/^207^Pb = 1.22), as is typical for well-mixed, not contaminated sediments, e.g., pre-1900 sediments from the Baltic Sea (Zaborska 2014). The model was based on the equation ^206^Pb/^207^Pb_mix_ = ^206^Pb/^207^Pb_A_(Pb_A_*f/Pb_mix_) + ^206^Pb/^207^Pb_L_*(Pb_L_*(1 − *f*)/Pb_mix_), where ^206^Pb/^207^Pb_mix_ and Pb_mix_ are the values for a mixture of the lithogenic (Pb_L_) and anthropogenic Pb (Pb_A_), and *f* is the proportion of the respective components. *Crosses on the modeled lines* mark 10 % steps in the mixing model

**Fig. 8:**
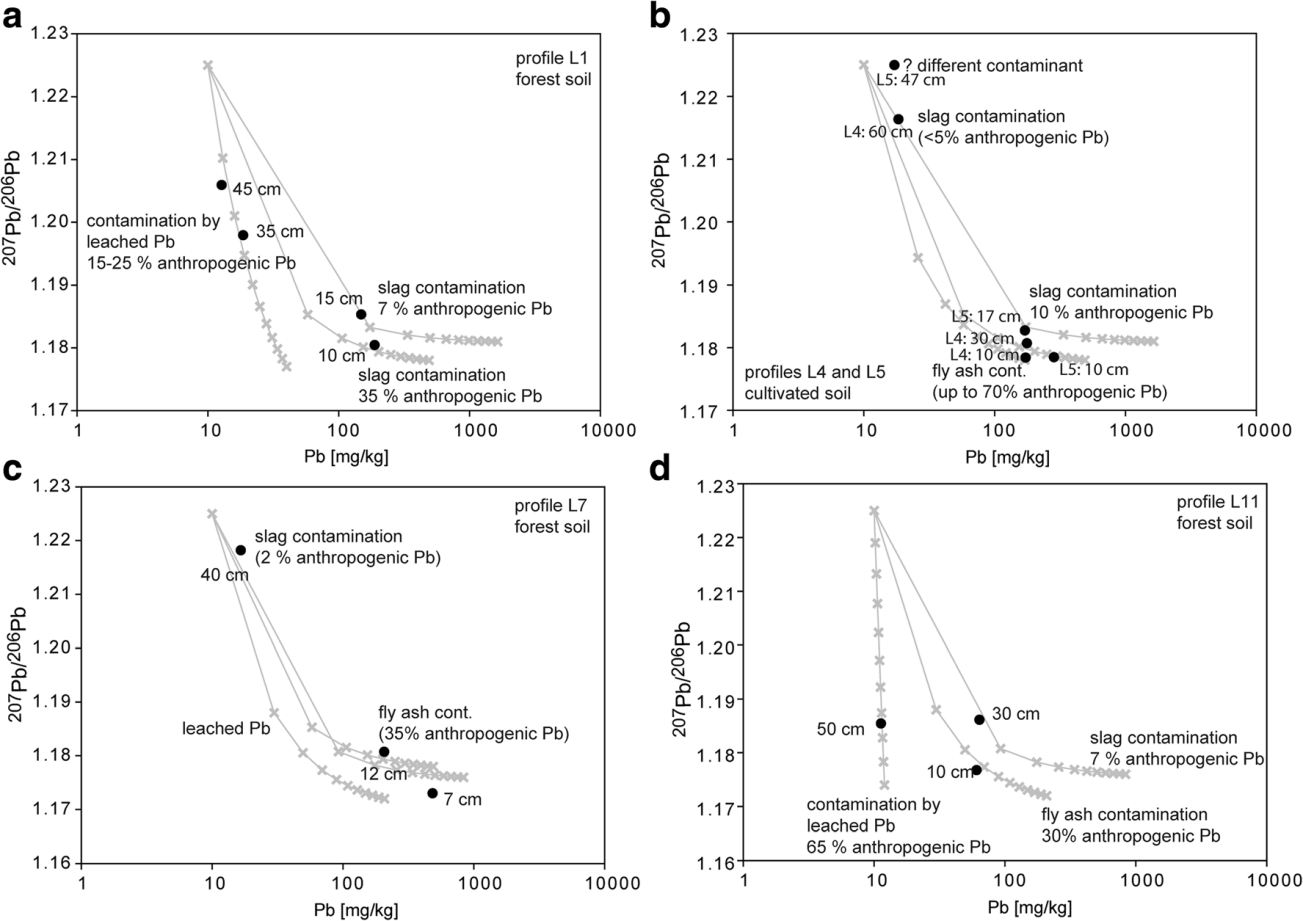
Mixing model as described in Fig. 7, presenting individual soil profiles. Approximate proportions of anthropogenic Pb and its source are indicated for each sample

The conclusions based on PTE concentrations are supported by Pb isotope ratios. Generally, Pb isotope ratios show that the upper soil horizons are also often dominated by anthropogenic Pb (lower ^206^Pb/^207^Pb ratios), compared with the lower soil horizons (Haack et al. 2003; Fernandez et al. 2008; Prapaipong et al. 2008) and that is the case also in this study. The high proportion of anthropogenic Pb in the upper soil horizon is also evidenced by the simple binary mixing models (Fig. 7). Despite uniform Pb isotope composition of smelting products emitted from the Legnica smelter, the soil samples show wide range of both Pb concentrations and Pb isotope compositions (Fig. 7). Such scatter is consistent with Pb being distributed through the soil profile in different forms and coming from components with different Pb concentrations. In detail, soils with high Pb concentrations and low Pb isotope ratios represent contamination dominated by Pb-rich slag particles (Fig. 7a), moderate Pb concentrations and low Pb isotope may represent predominately fly ash contamination (Fig. 7b), and low Pb concentrations and low Pb isotope ratios may mark contamination by soil solutions leached from the weathering of the slags and fly ashes (Fig. 7c). Variable Pb concentrations and similar Pb isotope ratios in contaminants result in soil profiles having distinct Pb isotope characteristic, even though Pb concentration always decreases with depth (Fig. 8). Generally, the upper soil horizon (<30 cm depth) could contain variable proportions of anthropogenic Pb from several to 100 %.

Interestingly, the lower soil horizons can also contain variable proportions of anthropogenic Pb from almost 0 to 65 %. Also, the Pb should commonly come from soil solutions (profiles L1 and L11, Fig. 8a, d). However, in some cases, the Pb isotope composition and Pb concentration in lower horizon can be explained by addition of smelter-emitted particles as in L7 profile representing forest soil and L4 and L5 profiles representing cultivated soils. Importantly, high proportions of anthropogenic Pb as soil solutions in lower horizons (up to 65 %) suggest that Pb moves or previously moved downwards the profile, but probably was not bound due to the lack of reactive surfaces (similar process was observed by Prapaipong et al. 2008). The outcome is that the isotope composition of the lower soil horizons is variable depending on the local availability of sorption sites. The implication is that Pb and other metal(oid)s may be constantly leached to the groundwater at unknown rate. One way to crosscheck the conclusions from Pb isotope modeling is to identify metal-bearing phases in different soil profiles.

### Primary and secondary PTE carriers: identification of phases

At sites contaminated by nearby smelting, metal(oid)s are mainly added to the soils as particles emitted from the smelter (Chopin and Alloway 2007). These particles are composed of dense phases and could be identified by the analyses of heavy mineral fraction. Also, the weathering products of the original particles should be present in the heavy mineral fraction. High anthropogenic Pb concentration in the upper soil horizon is consistent with Pb being located mostly in the original particles emitted from the smelter and their weathering products.

The SEM-EDS survey of the heavy mineral fraction was done in this study for one forest (L7) and one cultivated soil (L4), both contaminated by slag particles at depth according to Pb isotope modeling (Fig. 8). The survey showed that Pb, Zn, and Cu occurred in a variety of phases, characterized by different weathering rates. For example, Pb was the main constituent in metallic Pb droplets, which should weather quickly at the contact with atmosphere (e.g., Cao et al. 2003). However, the droplets observed in this study were mainly enclosed within fragments of Cu sulfides and/or silicate glass, i.e., the phases characterized by different weathering rates (Ettler et al. 2002; Kierczak et al. 2013), and their weathering was limited only to the inclusions in the rims. Similar behavior is shown by Cu, which formed Cu sulfides, and the sulfides were weathered and surrounded by secondary Fe oxides when in the direct contact with atmosphere (Fig. 5e) or were unweathered if enclosed in other phases (Fig. 4c, f). The transfer of Cu, Zn, and Pb from primary to secondary phases is evident as the metals were often detected within rims composed of secondary phases, but the rims have different origins. For example, Zn formed hemimorphite as a newly crystallized rim on the irregular Fe oxide, which was a secondary phase itself (Fig. 4e). On the other hand, Pb and Cu occurred in minor amounts in the Fe- and Si-rich rims, which were formed by direct weathering of primary sulfides and silicates (Fig. 5e, f). Complex, secondary Pb phases were also formed as separate grains (Fig. 5a). The general implication coming from the observations of heavy minerals is that metals such as Cu, Zn, and Pb were mobilized from their primary phases and bound in a variety of secondary phases either forming their own phases as hemimorphite and cerussite or being bound in Fe-rich phase. This Fe-rich phase, probably a Fe-(hydr)oxide as suggested by low totals in microprobe analyses, replaced both glass and sulfide, either whole surface (Fig. 4e, f) or at the rims and along the cracks (Fig. 5e, f). This phase was enriched in Cu, Zn, and Pb compared with smelter-emitted Fe-rich particles, as indicated in both EDS and microprobe analyses (Figs. 4f and 5f; Table 2). At the same time, some metal(oid)s were not redistributed and remained in the primary phases. For example, the Pb silicates were not affected by the weathering and they probably could remain in the soil as passive Pb carrier. Also, the complex inner structure of the particles and inclusions of one phase in the other show that leaching of metal(oid)s might have been delayed in time and controlled by weathering rate of slowly weathered phases. In summary, Pb and other metal(oid)s can remain in original particles or were transferred to secondary phases, which is consistent with Pb isotope modeling showing high proportion of anthropogenic Pb coming from slags and fly ashes in the upper soil horizon.

Interestingly, relatively large, unweatherd Pb silicates as well as slag fragments and fly ash spheres were detected at depths below 30 cm in both forest (L7) and cultivated soil (L4), also in agreement with Pb isotope modeling for these two profiles (Fig. 8). This shows that such large particles could be transported downward through the soil horizons probably due to soil cultivation and, therefore, mechanical mixing. The presence of such phases also in forest soils could be, because the current forest soils around the Legnica smelter were cultivated over 20 years when the smelter was active and only after this period the forested zone was formed.

### Potential environmental risks posed by polluted soils in Legnica

The studies assessing metal(oid) distribution and mobility in polluted soils are often based on chemical and Pb isotope composition of both soils and products of soil extractions and sequential extractions (e.g., Bacon and Hewitt 2005; Fernandez et al. 2008; Bove et al. 2011). Over 30 % of the Pb, Cd, and Cu in the first 30 cm of the soil in Legnica can be extracted by EDTA, suggesting relatively large mobility of these metals in the upper horizon and possibility of the their transfer to the lower soil horizons. Mobilized metals are mostly of anthropogenic origin as suggested by ^206^Pb/^207^Pb ratios in EDTA leachates typical for the products of Cu smelting. Interestingly, the metal(oid) load of the soils remains the same for over 30 years (Karczewska et al. 2010) despite the fact that the addition of the new material with high metal(oid) contents was vastly reduced in early 1990s (Monograph of KGHM). This together may indicate that the soils reached a semi-equilibrium state for the soil parameters prevailing now around the Legnica smelter (e.g., slightly acidic to slightly alkaline pH), and all available reactive sides for metal(oid)s are occupied. Therefore, we suggest that high proportions of anthropogenic Pb in some soils and low in others, e.g., below 30 cm depth, are rather due to lack of the reactive sites in the soils than due to slow transfer of the metal(oid)s along the soil profile. This is also consistent with Pb isotope modeling and high proportion of anthropogenic Pb in lower soil horizon coming predominately from soil solutions. In this context, we suggest that the high EDTA-extractable metal(oid) concentrations in the uppermost 30 cm of the soils indicate that most of the metal(oid)s is bound in secondary or primary phases prone to the weathering and these metal(oid)s can be potentially mobilized in the future. In detail, most of the anthropogenic metal loads were transferred from the original metal-bearing particles to metal-rich (e.g., cerussite, hemimorphite) or metal-bearing (e.g. Fe oxides/hydroxides) secondary phases such as those in Fig. 4e, f. This is consistent with (1) the general presence of Fe-oxy-hydroxides with elevated metal(oid) concentrations in contaminated soils (van Oort et al. 2006; Schroth et al. 2008) and (2) the results from sequential extractions indicating that Pb and other PTE, especially at contaminated sites, is rather bound in reducible not oxidizable fractions (e.g., Kabała and Signh 2001; Emmanuel and Erel 2002; Ettler et al. 2005; Bacon et al. 2006; Chrastný et al. 2012).

One additional complexity arises from comparing forest and cultivated soils. The L7 (forest) and L4 (cultivated land) profiles are different in terms of metal(oid) concentrations in EDTA leachates. General absence of reactive Pb phases at depths below 30 cm is suggested by lithogenic Pb isotope ratio in EDTA leachate from the forest soil. In contrast, the EDTA leachate from the deeper cultivated soil could contain approximately 10–20 % of anthropogenic Pb (based on the model in Fig. 7). This may be due to continuous transport of reactive particles to lower depths in cultivated soils, whereas similar transport stopped in the forest soils 40 years before our samples were taken, the time necessary for the reactive phases to be completely weathered and for Pb to be moved to lower depths.

Summarizing, despite seemingly simple metal(oid) distribution through the soil profiles around the Legnica smelter, the anthropogenic metal(oid)s are contained in variable primary phases emitted from the smelter, in the secondary phases derived from the weathering of the emitted particles as well as are transferred by soil solutions and probably bound by soil phases. This shows that the binding and mobility of metal(oid)s in polluted soils is a dynamic process and probably difficult to reconstruct in sites, where pollution load is isotopically variable.

## Conclusions

Soils around the Legnica copper smelter are characterized by high proportion of anthropogenic Pb and other metal(oid)s that are mostly limited to the upper 30 cm of the soil, both in the forest and cultivated soils. Lead isotope modeling is consistent with upper horizon being contaminated predominately by slag or fly ash particles whereas anthropogenic Pb in lower soil horizon is transferred in dissolved form. Therefore, the current distribution of metal(oid)s in Legnica soils is probably the function of (a) soil type in the way that cultivated soils may contain more reactive phases located deeper in soil profiles than forest soils, (b) presence or absence of slag in the soil profile since slag particles contain more metal(oid)s than fly ashes, and (c) the availability of binding sites for anthropogenic Pb moving down the soil profile. However, in general, the distribution of all metal(oid)s with depth is similar between the analyzed soil profiles. This similarity is surprising because metals such as Cu, Zn, and Pb were detected in a variety of phases emitted from the smelter and these phases weathered at different rates. Therefore, this general uniformity shows that the distribution of metal(oid)s through the soil profiles is probably due to saturation of the reactive sites and we believe that metal(oid)s could move through the lower horizons via unreactive pathways and may reach and contaminate groundwaters. This is consistent with relatively constant metal(oid) contents in the soils surrounding the Legnica smelter for over 30 years, despite vast reduction in emissions of metal(oid)s from the smelter.

The metal(oid)s may be transported downward in dissolved and colloidal forms. However, another way to move metal(oid)s downward the soil profile is via the transport of larger (50–100 μm in diameter) particles in cultivated soils. Interestingly, the lower horizons in the currently cultivated soils contain both unweathered and weathered particles, whereas the weathered ones are absent in the forest soils. This is consistent with the weathered particles being removed from the forest soils after an extensive period of weathering, i.e., around 40 years that passed since the soils were forested and turned into the protection zone. The scattered Pb isotope composition in the lower soil horizons may be, therefore, the result of some of the Pb being bound to local sites, or it may be also due to the transport of larger, Pb-rich, unreactive particles through the soil profile. Therefore, our results show that metal(oid)s mobility in the contaminated soils is a complex dynamic process and relatively simple distribution of metal(oid)s in soils may be due to saturation of the available sorption sites, after which the metal(oid)s moves freely through the soil profile.

## Electronic supplementary material

ESM 1 (DOCX 13 kb)

ESM 2 (XLSX 10 kb)
