# Supplementary material for: Lead isotopes and heavy minerals analyzed as tools to understand the distribution of lead and other potentially toxic elements in soils contaminated by Cu smelting (Legnica, Poland)
Source: Environ Sci Pollut Res Int. 2016 Sep 21;23(23):24350–63. doi: 10.1007/s11356-016-7655-4 (PMC5110706; doi:10.1007/s11356-016-7655-4)
Supplement: Supplementary file 1 — (DOCX 13 kb) [file 11356_2016_7655_MOESM1_ESM.docx]

| Table S1 The general characteristic of the vegetation and soils in the Legnica area. | | | | |
| --- | --- | --- | --- | --- |
| Sample number | GPS locality | Type of flora | Litter | Type of soil |
| 1 HML | N 51 18 60,1  W 16 10 55,8 | Populus canescens, Betula pendula, Calamagrostis epigeios. | Moder | Cutanic Luvisol |
| 2 HML | N 51 19 55,9  W 16 10 31,8 | Populus canescens, Betula pendula, Calamagrostis epigeios , Solidago gigantea,  Festuca rubra, Senecio vulgaris, Anthriscus sylvestris, Taraxacum officinale, Trifolium repens. | Mull | Cutanic Luvisol |
| 3 HML | N 51 20 17,2  W 16 10 09,5 | Populus canescens, Betula pendula, Calamagrostis epigeios, Solidago gigantea,Senecio vulgaris, Anthriscus sylvestris, Taraxacum officinale, Artemisia vulgaris | Mull | Cutanic Luvisol |
| 4 HML | S 51 18 63,4  E 16 12 91,0 | Populus canescens, Betula pendula, Calamagrostis epigeios, Solidago gigantea,Senecio vulgaris, Anthriscus sylvestris, Taraxacum officinale, | Moder | Cutanic Luvisol |
| 5 HML | S 51 18 46,1  E 16 13 63,1 | Populus canescens, Betula pendula, Calamagrostis epigeios, Solidago gigantea,Senecio vulgaris, Taraxacum officinale, | Mull | Cutanic Luvisol |
| 6 HML | S 51 18 38,6  E 16 14 12,9 | Populus canescens, Betula pendula, Calamagrostis epigeios, Senecio vulgaris, | Mull | Cutanic Luvisol |
| 7 HML | S 51 17 83,7  W 16 10 59,2 | Populus canescens, Betula pendula, Calamagrostis epigeios, Solidago gigantea, Potentilla anserina | Mull | Cutanic Luvisol |
| 8 HML | S 51 17 32,1  W 16 11 09,1 | Populus canescens, Betula pendula, Calamagrostis epigeios, Solidago gigantea, Festuca pratensis Huds., Matricaria chamomilla | Mull | Cutanic Luvisol |
